# Characterization of gut microbiota associated with metabolic syndrome and type-2 diabetes mellitus in Mexican pediatric subjects

**DOI:** 10.1186/s12887-023-03983-6

**Published:** 2023-05-03

**Authors:** Ana K. Carrizales-Sánchez, Oscar Tamez-Rivera, Nora A. Rodríguez-Gutiérrez, Leticia Elizondo-Montemayor, Misael Sebastián Gradilla-Hernández, Gerardo García-Rivas, Adriana Pacheco, Carolina Senés-Guerrero

**Affiliations:** 1 Tecnologico de Monterrey, Escuela de Medicina y Ciencias de La Salud, Av. Ignacio Morones Prieto 3000, Monterrey, Nuevo Leon C.P. 64710 Mexico; 2 Tecnologico de Monterrey, Escuela de Ingenieria y Ciencias, Av. Eugenio Garza Sada 2501 Sur, Monterrey, Nuevo Leon C.P. 64849 Mexico; 3 Hospital Regional Materno Infantil de Alta Especialidad, Av. San Rafael 460, C.P. 67140 Guadalupe, Nuevo Leon Mexico; 4 Tecnologico de Monterrey, Escuela de Ingenieria y Ciencias, Av. General Ramon Corona 2514, Zapopan, Jalisco C.P. 45138 Mexico; 5 Tecnologico de Monterrey, The Institute for Obesity Research, Av. Eugenio Garza Sada 2501 Sur, C.P. 64849 Monterrey, Nuevo Leon, Mexico

**Keywords:** Gut microbiota, Child, Adolescent, Diabetes Mellitus, Type II, Metabolic Syndrome X

## Abstract

**Background:**

Childhood obesity is a serious public health concern that confers a greater risk of developing important comorbidities such as MetS and T2DM. Recent studies evidence that gut microbiota may be a contributing factor; however, only few studies exist in school-age children. Understanding the potential role of gut microbiota in MetS and T2DM pathophysiology from early stages of life might contribute to innovative gut microbiome-based interventions that may improve public health. The main objective of the present study was to characterize and compare gut bacteria of T2DM and MetS children against control subjects and determine which microorganisms might be potentially related with cardiometabolic risk factors to propose gut microbial biomarkers that characterize these conditions for future development of pre-diagnostic tools.

**Results:**

Stool samples from 21 children with T2DM, 25 with MetS, and 20 controls (*n* = 66) were collected and processed to conduct 16S rDNA gene sequencing. α- and β-diversity were studied to detect microbial differences among studied groups. Spearman correlation was used to analyze possible associations between gut microbiota and cardiometabolic risk factors, and linear discriminant analyses (LDA) were conducted to determine potential gut bacterial biomarkers. T2DM and MetS showed significant changes in their gut microbiota at genus and family level. Read relative abundance of *Faecalibacterium* and *Oscillospora* was significantly higher in MetS and an increasing trend of *Prevotella* and *Dorea* was observed from the control group towards T2DM. Positive correlations were found between *Prevotella*, *Dorea*, *Faecalibacterium*, and *Lactobacillus* with hypertension, abdominal obesity, high glucose levels, and high triglyceride levels. LDA demonstrated the relevance of studying least abundant microbial communities to find specific microbial communities that were characteristic of each studied health condition.

**Conclusions:**

Gut microbiota was different at family and genus taxonomic levels among controls, MetS, and T2DM study groups within children from 7 to 17 years old, and some communities seemed to be correlated with relevant subjects’ metadata. LDA helped to find potential microbial biomarkers, providing new insights regarding pediatric gut microbiota and its possible use in the future development of gut microbiome-based predictive algorithms.

**Supplementary Information:**

The online version contains supplementary material available at 10.1186/s12887-023-03983-6.

## Background

Childhood obesity is considered by the World Health Organization as one of the most severe public health challenges in the twenty-first century [1] and has been linked as a precursor of relevant metabolic disorders such as metabolic syndrome (MetS) and type-2 diabetes mellitus (T2DM) [2]. Obesity had a global tenfold increase in its prevalence within school-age children during the last four decades due to deficient nutritional habits and sedentarism, disregarding their socioeconomic status [3, 4]. The pathophysiology of childhood obesity and MetS has not been completely elucidated; however, it is well known that an interaction between genetic and environmental factors may be contributing to it [5]. Insulin resistance (IR) is one of the risk factors that characterize MetS, which is promoted by an abnormal accumulation of adipose tissue through the activation of various inflammatory mechanisms, such as adipokine deregulation and an increase in free fatty acid release, affecting the functionality of relevant organs such as the pancreas, liver, skeletal muscle, and adipocytes [6]. This trait is also associated with a greater risk of developing T2DM, which just in the United States has shown an annual increase of 4.8% among children and adolescents compared to a 1.9% rise in the type-1 variant [7]. Meanwhile, in Mexico, the prevalence of T2DM in children from 10 to 19 years old in the period of 2014 to 2019 has increased 34%, approximately [8].

Gut microbiota has shown to be implicated in multiple human metabolic processes that may affect their carbohydrate, lipid, and amino acid metabolism or even trigger immune and inflammatory responses [9]. Alterations in its composition have been associated with relevant metabolic diseases such as obesity and diabetes [10]. Turnbaugh *et al *[11] described an obesogenic genotype of the gut microbiota by comparing obese and lean mice where traits of a higher abundance of the phylum Firmicutes and a decrease of Bacteroidetes were observed. Interestingly, when gut microbiota of obese mice was transplanted to germ-free mice, the latter adopted the donor’s phenotype, displaying an increase in adiposity and triglyceride levels, and developed insulin resistance, independently of their food regime [11, 12].

During the last years, studies regarding gut microbial communities have gained popularity but efforts have been mostly focused on human adults than within the pediatric population, possibly due to ethical concerns and problems associated with retrieving samples [13]. This study provides valuable information to lay the foundations of gut microbiota research in pediatric subjects and identify possible factors associated with developing MetS and T2DM.

## Results

### Metadata statistics

A total of 66 subjects from 7 to 17 years old with T2DM (*n *= 21), MetS (*n* = 25) and healthy controls (*n* = 20) conformed the studied population where 53% were male and 47% female. Anthropometric, biochemical, clinical, and demographic characteristics are shown in Table 1. As expected, the control group presented normal metabolic parameters. Weight, BMI, waist circumference, TG levels, and plasma fasting blood glucose showed to be significantly higher in MetS (58.70 kg, 25.60 kg/m^2^, 85.30 cm, 144.00 mg/dL, 96.00 mg/dL, respectively) and T2DM groups (67.10 kg, 27.90 kg/m^2^, 93.00 cm, 145.00 mg/dL, 115.00 mg/dL, respectively) compared to the control (34.25 kg, 17.35 kg/m^2^, 62.00 cm, 87.50 mg/dL, 84.50 mg/dL, respectively), but they also demonstrated to be significantly higher in the T2DM individuals compared to MetS group.

**Table 1:** Anthropometric, biochemical, clinical, and demographic features from the studied population (*n* = 66)

	**Control (*n* = 20)**	**MetS (*n* = 25)**	**T2DM (*n* = 21)**	**p-value**
**Age (years)**	10.50 (9.00—13.00) ***b***	12.00 (10.00—14.00) ***c***	14.00 (13.00—15.00) ***bc***	< 0.001*
**Males / Females (n)**	11/9	14/11	10/11	0.863
**Height (cm)**	141.00 (133.00—158.25) ***b***	153.00 (146.00—164.00)	159.00 (155.00—165.00) ***b***	0.003*
**Weight (kg)**	34.25 (27.50—44.20) ***ab***	58.70 (50.90—79.30) ***ac***	67.10 (61.40—87.40) ***bc***	< 0.001*
**BMI (kg/m**^**2**^**)**	17.35 (15.88—18.03) ***ab***	25.60 (23.30—29.40) ***ac***	27.90 (25.70—31.40) ***bc***	< 0.001*
**Waist Circumference (cm)**	62.00 (57.63—68.28) ***ab***	85.30 (79.00—93.30) ***ac***	93.00 (85.50—102.00) ***bc***	< 0.001*
**Systolic BP (Percentile)**	37.50 (26.75—40.50) ***b***	62.00 (28.00—83.00)	62.00 (32.00—90.00) ***b***	0.048*
**Diastolic BP (Percentile)**	63.00 (57.00—66.00)	56.00 (39.00—73.00)	74.00 (50.00—94.00)	0.395
**TC (mg/dL)**	154.35 ± 21.45	157.96 ± 32.73	150.81 ± 29.44	0.095
**TG (mg/dL)**	87.50 (65.75—96.50) ***ab***	144.00 (123.00—201.00) ***ac***	145.00 (112.00—181.00) ***bc***	< 0.001*
**HDL-c (mg/dL)**	53.00 (43.75—59.75) ***ab***	38.00 (35.00—40.00) ***a***	39.00 (32.00—44.00) ***b***	< 0.001*
**LDL-c (mg/dL)**	99.35 ± 22.22 ***b***	82.29 ± 26.83	76.95 ± 20.87 ***b***	0.010*
**Glucose (mg/dL)**^†^	84.50 (79.25—87.50) ***ab***	96.00 (90.0—103.00) ***ac***	115.00 (92.00—177.50) ***bc***	< 0.001*

Data are shown as mean ± SD for parametric data and median (Q25%—Q75%) for nonparametric data. Significance (“*”) was established as *p*-value ≤ 0.05 where one-way ANOVA was used for parametric variables, Kruskall-Wallis test for nonparametric variables, and Fisher’s exact test for nominal binary variables. Interactions among study groups were analyzed using Tukey HSD test for parametric data and Dunn’s test for nonparametric data. ***a*** represents statistical difference between control and MetS, ***b*** between control and T2DM, and ***c*** between MetS and T2DM; ^†^8 h fasting

Additionally, HDL-c levels showed to be significantly lower in MetS (38.00 mg/dL) and T2DM (39.00 mg/dL) compared to the control group (53.00 mg/dL) and, interestingly, LDL-c levels seemed to be significantly lower in the T2DM group (76.95 ± 20.87 mg/dL) compared to the control (99.35 ± 22.22 mg/dL). TC levels did not show to be different among the studied groups. In the case of blood pressure (BP) parameters, a significantly higher systolic BP percentile was found in the T2DM group (62.00) compared to the control group (37.50). The MetS group got the same median value of systolic BP percentile (62.0) as T2DM group but with no statistical significance when compared to the controls. Moreover, the diastolic BP percentile did not demonstrate significance among the studied groups.

Statistical analysis showed that abdominal obesity was present in 71% (15/21) and 80% (20/25) of children with T2DM and MetS, respectively, whereas 19% (4/21) and 20% (5/25) of them were overweight (Additional File 1—Table S1). Risk factors that characterize MetS such as abdominal obesity, high TG levels, low HDL-c, and high plasma fasting glucose levels were significantly more prevalent in the MetS and T2DM individuals compared to the control group; meanwhile, high BP was exclusively significant in the T2DM group compared to the controls (Additional File 1—Table S1).

Noteworthy, clinical evaluation surveys also demonstrated that most of the MetS and T2DM subjects were under medical treatment (Additional File 1—Table S2): 88% of the MetS group were taking metformin where significant differences were found between the control group and MetS and T2DM individuals, as well as MetS compared to T2DM individuals. Meanwhile the rest of the studied individuals (12%) were following a diet + exercise regime. Finally, 57% of the T2DM group were taking metformin, 29% metformin + insulin, 9.5% insulin, and 4.5% a diet + exercise regime. There was a significant difference between the control group and T2DM individuals and the MetS group compared to T2DM individuals that were taking metformin + insulin.

### Taxonomic characterization, and alpha and beta diversity

Sequencing of sample libraries resulted in around 8.4 and 16.5 million reads passing quality filters per study group (individual reads per sample are shown in Additional File 1—Table S3; total OTUs from the studied groups are shown in Additional File 1—Table S4). Rarefaction curves indicate that sufficient sampling was achieved (Additional File 2—Figure S1A and Figure S1B). α-diversity was determined by calculating richness, Chao1, Shannon, and Simpson indices for each study group. Richness showed no statistical significant differences among groups (Additional File 2—Figure S2A). Moreover, Chao1 index also demonstrated the same behavior with no statistical significance (Additional File 2—Figure S2B).

Shannon index (Additional File 2—Figure S2C) was significantly higher in the MetS group compared to the controls but not with the T2DM study group. Finally, Simpson index exhibited to be significantly higher in the MetS and T2DM study groups compared to the controls (Additional File 2—Figure S2D).

Relative abundance of gut bacterial communities and clustering by PCoA were analyzed at phylum (Additional File 1—Table S5), family (Additional File 1—Table S6), and genus (Additional File 1—Table S7) taxonomic levels to study β-diversity of these bacterial communities. The gut microbiome in all studied groups was mainly composed by two major phyla: Bacteroidetes and Firmicutes (Additional File 2—Figure S3A). Firmicutes dominated over Bacteroidetes but differences between these phyla were more pronounced in MetS (69 and 28% for Firmicutes and Bacteroidetes, respectively) compared to the T2DM (61 and 35%) and the control group (57 and 41%). However, PCoA analysis did not show significant differences among studied groups at this taxonomic level (Additional File 2—Figure S3B).

At family level, the gut environment was dominated by Bacteroidaceae, Lachnospiraceae and Ruminococcaceae (Additional File 2—Figure S4A). Bacteroidaceae was the major family in the control group belonging to the phylum Bacteroidetes, while MetS and T2DM showed a higher abundance of Lachnospiraceae and Ruminococcaceae, both belonging to Firmicutes. T2DM had particularly the presence of the Succinibrionaceae family, belonging to Proteobacteria, which was not detected in the predominant bacteria from the other two study groups. Moreover, PCoA analysis showed that the T2DM study group was significantly different compared to the MetS and control groups but there was no significant difference between the MetS subjects with the controls (Additional File 2—Figure S4B).

**Fig. 1:**
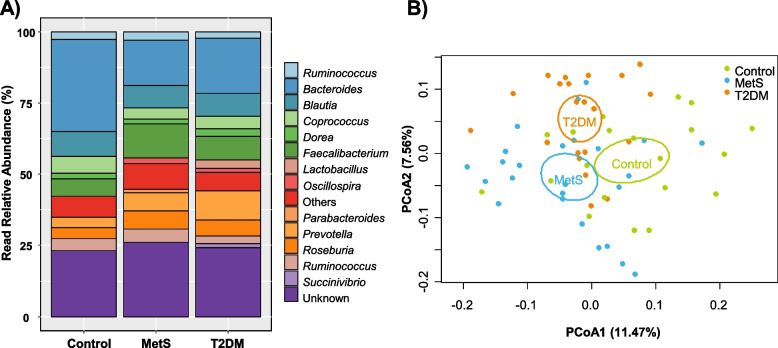
Read relative abundance (**A**) and clustering by PCoA (**B**) of gut microbiota genera. Data represent read relative abundances greater or equal to 1%. Each dot corresponds to an individual’s bacterial community in a specific group

Moreover, the most numerous bacteria at genus level in all studied groups were *Bacteroides*, *Faecalibacterium* and *Blautia* (Fig. 1A). *Bacteroides* was the dominant genus in the control group (32% relative abundance), which was represented 2.0-fold higher than in the MetS and T2DM groups. As the second most abundant genus in the control group, *Blautia* exhibited similar values among all groups (8–9% read relative abundance). On the contrary, *Faecalibacterium* was more abundant in the MetS and T2DM groups than in the control by 1.9 and 1.3-fold, respectively.

Another particularly abundant genus in MetS and T2DM was *Prevotella* with an abundance of 7 and 11%, respectively, while the control showed 3.8% abundance. Of all the studied groups, T2DM showed two genera in low abundances that were present exclusively in this group: i.e., *Lactobacillus* (3%) and *Succinivibrio* (2%). All studied microbial communities showed similar values of the group “Others” (5–8%) represented by ≤ 1%, and the group “Unknowns” (23–26%). Finally, PCoA at this taxonomic level showed a clear dissimilar pattern among groups, demonstrating that the gut microbiota of children with MetS, T2DM and control was different at the genus level (Fig. 1B).

### Statistical analyses of relevant communities at genus level

**Fig. 2:**
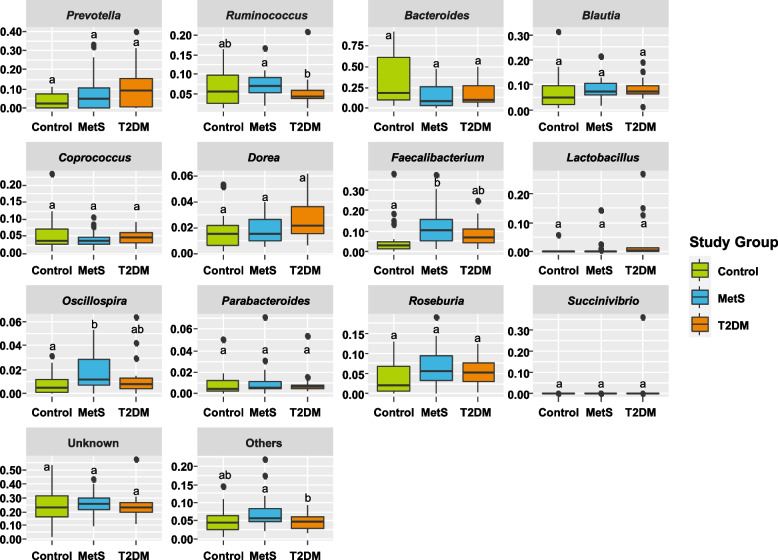
Comparison of relevant genera read relative abundance at each study group. Kruskall-Wallis test was conducted, and Dunn test was used when the p-value was ≤ 0.05 among study groups, using read relative abundance as dependent variable. The “Others” category belongs to all microbial communities with a relative abundance ≤ 1% within the study group. Study groups sharing the same letter (a or b) are not significantly different among them (p-value > 0.05)

Bacteria at genus level with read relative abundance > 1% were selected to conduct Kruskall-Wallis tests to determine if their presence were significantly different among the three studied groups (Fig. 2; Additional File 1—Table S8). *Faecalibacterium* and *Oscillospira* (Ruminococcaceae family and Firmicutes phylum), showed to be significantly higher in MetS individuals compared to the control group (p-value = 0.003 and 0.018, respectively). *Ruminococcus* (Ruminococcaceae family and Firmicutes phylum) demonstrated to have a significantly lower presence in the T2DM condition compared to the MetS group (p-value = 0.036). Noteworthy, read relative abundance of “Others” also showed to be significantly higher in the MetS group compared to the T2DM groups but with no statistical significance compared to the control individuals (p-value = 0.031).

*Prevotella, Dorea, Roseburia,* and *Blautia* did not show a significant difference in their abundance among study groups. However, *Prevotella* (Prevotellaceae family and Bacteroidetes phylum) and *Dorea* (Lachnospiraceae family and Firmicutes phylum) showed to increase from the control to a T2DM state, while *Roseburia* and *Blautia* (Lachnospiraceae family and Firmicutes phylum) showed to be lower in the control group compared to the MetS and T2DM conditions. In the case of *Bacteroides* (Bacteroidaceae family and Bacteroidetes phylum) and *Parabacteroides* (Porphyromonadaceae family and Bacteroidetes phylum), the first one showed to be higher in the control group compared to MetS and T2DM conditions but with no statistical significance (p-value = 0.070) and the second one remained stable within the three study groups. Finally, very low reads were detected for *Lactobacillus* (Lactobacilliaceae family and Firmicutes phylum) and *Succinivibrio* (Succinivibrionaceae family and Proteobacteria phylum).

### Correlational studies with subjects’ metadata and their taxonomic composition

**Fig. 3:**
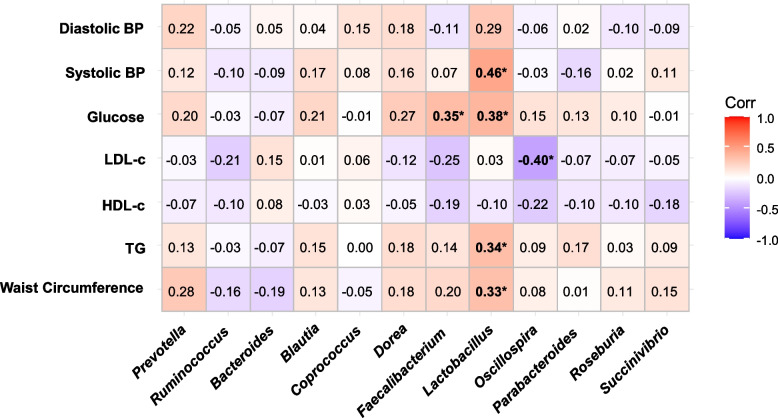
Correlation of relevant genera read relative abundance at each study group with clinical metadata. Spearman correlation test was conducted, where “*” indicates statistical significance with a corrected p-value using the Bejamini-Hochberg procedure (corrected p-value ≤ 0.05)

Spearman correlations (p-values were corrected with the Bejamini-Hochberg procedure) were performed to analyze correlations between the most abundant gut bacteria at the genus level and biochemical and anthropometric variables related to cardiometabolic risk factors that characterize MetS, including LDL-c levels (Fig. 3). Interestingly, *Lactobacillus* displayed the most significant positive correlations with the subjects’ metadata: systolic BP (0.46), glucose levels (0.38), waist circumference (0.33), and triglyceride levels (0.34). Additionally, *Faecalibacterium* displayed a significant positive correlation with glucose levels (0.35), while *Oscillospira* demonstrated a significant negative correlation with LDL-c levels (-0.40).

### Linear discriminant analysis using gut microbial communities as predictive tool

Gut bacterial differences among MetS, T2DM, and controls were analyzed through LDA using a data set comprising the relative abundance of 13 families and 21 genera whose abundance was > 1% in each of the studied individuals and were found to display significant differences between the groups of individuals after conducting one-way ANOVA analyses and correcting the p-value with the Bejamini-Hochberg procedure (corrected p-value ≤ 0.05). Two linear discriminant functions (LD1 and LD2) were determined, and the standardized coefficients were retrieved for each bacterium at each taxonomic level (family and genus).

**Fig. 4:**
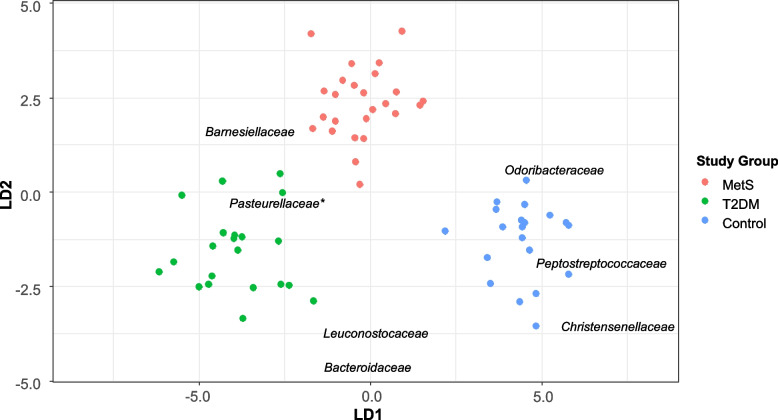
Linear discriminant analysis (LDA) from gut bacterial communities at family level from the studied groups

Six bacterial families found to significantly contribute to classify the individuals in their corresponding group (Fig. 4). Odoribacteriaceae (LD1 coefficient = 1.8), Peptostreptococcaceae (LD1 coefficient = 2.25), and Christensenellaceae (LD1 coefficient = 2.72) were highly associated with the control group while Barnesiellaceae (LD1 coefficient = -1.13) was found to be a potential indicator of MetS. Likewise, Leuconostocaceae and Bacteroidaceae (LD2 coefficients = -1.22 and -1.52) were inversely associated with a MetS state. Similarly*,* Pasteurellaceae was associated with T2DM and is thus regarded as a potential indicator of this condition, but the absolute standardized coefficient was lower than 1 (LD1 coefficient = -0.92) (Additional File 1—Table S9).

Moreover, at genus level (Additional File 2—Figure S4), *Acidaminococcus* was found to be an indicator of a T2DM condition (LD1 coefficient = 1.14) while *Bilophila* was more associated with a MetS state (LD1 and LD2 coefficients = -1.65 and -1.10). Likewise, *Bulleidia* (LD1 coefficient = 0.74) was highly associated with T2DM, *Clostridium* (LD2 coefficient = 0.73) with the control group, and *Succinispira* (LD1 and LD2 coefficients = -0.73 and -0.82) with MetS. However, their absolute standardized coefficients did not reach the 1 threshold (Additional File 1—Table S10).

## Discussion

In this work we observed that the gut bacterial diversity increased in MetS and T2DM compared to the control group. There are no reported studies regarding gut microbiome in school-age children with T2DM and there are just scarce reports in adults with MetS where most of them belong to studies in Mexican individuals. Nirmalkar *et al *[14] reported a similar result in Mexican pediatric subjects (12—18 years old) where bacterial diversity of the gut microbiome showed to be slightly higher in obese children and adolescents compared to normal weight individuals but with no statistical significance. Besides, studies in Mexican women with obesity and MetS also demonstrated to have a higher microbial diversity compared to the controls but not as high as individuals having exclusively an obese state [15], again, with no statistical significance.

Analyzing β-diversity, gut microbiota was different at family and genus levels among the studied groups, suggesting that bacteria may be implicated in these health conditions. By analyzing specific microorganisms, abundant genera (*Faecalibacterium*, *Oscillospira*, and *Ruminococcus*) belonging to the Ruminococcaceae family were significantly different among the studied groups. *Faecalibacterium* showed to be significantly higher in the MetS group compared to the controls but with no significant difference compared with the T2DM individuals. In addition, this genus also showed a weak but significant and positive correlation with glucose levels and negative with LDL-c levels. *Faecalibacterium* is a butyrate-producer and potential alleviator of T2DM [16], contradicting what was found in this study. However, other researchers [17, 18] have also reported a significantly increased abundance of this genus in obese Mexican children. In obese adult Chinese subjects, a negative correlation of this genus with LDL-c levels was also reported [19].

*Oscillospira* was also significantly higher in the MetS group compared to the controls but with no difference compared to T2DM individuals. Additionally, it showed a significantly moderate negative correlation with LDL-c levels. Species belonging to this genus have been found to be butyrate-producer and related with leanness [20], being able to confer benefits to its host. High abundance of *Oscillospira* seems to be influenced by exogenous factors such as probiotics, prebiotics, natural products, exercise, and diet [21]. Contrastingly to what it was found in this work, studies in South Korean adults showed that this genus was negatively but weakly correlated with a metabolic unhealthy status [22] and also this bacterial community has been reported to be positively correlated in the development of diabetes and inflammation in T2DM mice administered with high fat diets [23].

Finally, *Ruminococcus* significantly decreased in the T2DM group compared to the other studied individuals but with no significant correlation with cardiometabolic risk factors. Previous studies have related its abundance with T2DM [24, 25] but research conducted in T2DM individuals taking metformin have revealed that several species belonging to this genus seem to decrease in presence of this treatment [26]. It must be highlighted that in this study the clinical surveys performed to the studied subjects showed that most of the MetS and T2DM individuals were under medication. Metformin was taken by ~ 51.5% of the whole studied population, something that might be impacting the gut microbial composition and imperative to evaluate in further studies.

*Prevotella* (Prevotellaceae family), *Dorea* (Lachnospiraceae family), and *Lactobacillus* (Lactobacilliaceae family) were also abundant genera that did not show a significant difference among studied groups but that had a significant correlation with clinical metadata: *Prevotella* and *Dorea* increased their abundance from a healthy state to a T2DM condition but with no significant difference among groups. Moreover, statistical studies showed a weak but significantly positive correlation of *Dorea* with waist circumference, which is a parameter used to determine abdominal obesity for MetS. It has been previously reported a possible microbial co-occurrence between *Prevotella* and *Dorea* in an adult US Hispanic/Latino population, where *Prevotella* may be more associated with abdominal obesity due to its positive correlation with *Dorea*’s abundance, which was positively associated with BMI [27] and with obesity [28, 29]. Conversely, a study conducted in Korean adolescents reported a direct positive correlation of *Prevotella* with BMI [30].

On the other hand, it has been broadly reported that *Prevotella* may be negatively correlated with obesity, but recent studies have also found the probability that this genus’ abundance and its positive correlation with obesity may be more related to non-Hispanic white populations and/or people with high socioeconomic status. Meanwhile, the correlation between higher presence of *Prevotella* with obesity may be stronger within black and Hispanic individuals [31].

In the case of *Lactobacillus*, it has been positively correlated with waist circumference, TG levels, glucose levels, and both systolic and diastolic blood pressure parameters. It is well known that several species belonging to this genus (i.e., *Lactobacillus fermentum* and *L. plantarum*) have been broadly reported to have probiotic properties [32–34], nonetheless, other species like *L. reuteri* have been positively correlated with obesity and diabetes. A study in Mexican children that had a high-tertile fructose intake demonstrated that this species was positively correlated with BMI, waist circumference, TG levels, insulin concentrations, HOMA-IR, and low HDL-c levels [35]. Noteworthy, there are also studies that correlate increased *Lactobacillus* presence with metformin medication [36, 37].

Abundance from genera belonging to the Ruminococcaceae and Lachnospiraceae families seemed to be highly relevant while studying their relationship with each of the evaluated health conditions and clinical metadata. The presence of both families has been attributed to a healthy condition due to their ability to degrade complex dietary plant materials indigestible by the host [38], contrary to what was found herein. Moreover, studies conducted in T2DM Chinese adults evidenced that both families showed to be higher in a T2DM condition compared to the controls [39] meanwhile this was also observed in Mexican obese women and obese with MetS phenotypes [15]. On the other hand, studies conducted in atherosclerotic cardiovascular disease risk adult volunteers showed an increase in both families after a one-week intervention with 100% plant-based diet, stress management education, and exercise [40], showing that changes in lifestyle may also influence their richness. In this study, information regarding participants’ diet and lifestyle was not recorded, which is something that in the future may be considered in the study of these health conditions.

Finally, LDA was helpful to identify bacterial biomarkers that may not be within the most abundant microbial communities. Odoribacteraceae, Peptostreptococcaceae, and Christenellaceae were the most significant indicators of a healthy state: Odoribacteriaceae and Christenellaceae were previously reported to be more abundant in long-lived Chinese elderly, attributing them to longevity [41]. Odoribacteriaceae has also been inversely correlated with obesity in Caucasian adults [42] and Christenellaceae was found to be more abundant not just in normal-weight Mexican children [43] but also in metabolically healthy obese Chinese children [44]. Lastly, Peptostreptococcaceae has been reported to be more abundant in Filipino children who had a higher-energy fiber intake compared to a group with a lower fiber intake [45] but interestingly it also seemed to be more abundant in Mexican obese children compared to the normal-weighted ones [14], contrasting to what was found in the present study.

Moreover, in this study, Leuconostocaceae and Bacteroidaceae were negatively associated with a MetS state. Leuconostocaceae has been reported to be positively correlated with a metabolically healthy status in South Korean adults [22] and its increased abundance together with an increased abundance in Peptostreptococcaceae have been positively correlated with Italian obese adults with T2DM taking a Ma-Pi 2 diet, which comprises of a fiber-rich macrobiotic diet with a prebiotic potential [46]. Meanwhile, Bacteroidaceae has been regarded as a bacterial predictor of weight loss in short-term low-carbohydrate diet intervention within a Chinese adult cohort with obesity [47] and negatively correlated with an obese state in high fat diet (HFD)-induced obesity mice [48].

At genus level, *Acidaminococcus* and *Bilophila* were significantly associated with T2DM and MetS respectively in the present study. Furthermore, *Acidaminococcus* has been previously reported to be significantly and positively correlated with fasting glucose, also demonstrating a significant abundance in T2DM Indian adults compared to nondiabetic ones [49]. Moreover, *Bilophila* has been associated with higher states of inflammation, alterations in bile acid metabolism, and intestinal barrier dysfunction that may result in hepatic steatosis and glucose alterations in HFD male C57BL/6 J mice [50]. Additionally, this genus was found to be in Male Tsumura Suzuki obese diabetes mice compared to control mice which lacked its presence, relating this bacterial group with the onset of colitis [51].

It is known that the human gut microbiota develops throughout time; however, a relative stability in its composition is reached by school age [52, 53]. Because of this, we infer that the age range in this study may not have influenced the differences in bacterial diversity among the studied groups. With all the above-mentioned results, it has been seen that detecting key differences in the gut microbiota of the present studied groups could be used in the further development of predicting tools to detect the health conditions herein studied.

## Conclusion

Gut microbiota was different at family and genus taxonomic levels among controls, MetS, and T2DM study groups within children from 7 to 17 years old. Statistical analysis using the most abundant genera from each group revealed that *Faecalibacterium* and *Oscillospira* were significantly higher in the MetS groups compared to the T2DM individuals and controls, meanwhile *Ruminococcus* was significantly lower in T2DM compared to the MetS group. Positive correlations were found comparing *Prevotella*, *Dorea*, *Faecalibacterium*, and *Lactobacillus* with relevant cardiometabolic risk factors (hypertension, abdominal obesity, high glucose levels, and high triglyceride levels). Moreover, LDA revealed that analyzing the least abundant bacterial communities may be relevant in the discovery of microbial biomarkers that could be used for the future development of gut microbiome-based predictive algorithms.

Main limitations that must be considered of this work are the small population size and that most of the studied subjects within the MetS and T2DM conditions were under medical treatment, something that has already been reported to have an influence in the gut microbial composition. However, it is one of the first studies in school-age children with a confirmed diagnosis of MetS and T2DM, where working with high sample size is challenging due to sample obtention from pediatric subjects. Even though a targeted metagenomics study is limited to taxonomic characterizations, this study serves as a base for shotgun metagenomic studies. In the future, these data could be valuable to explore microbial metabolic routes implicated in children's dysbiosis, identify risk factors in developing T2DM and MetS, and contribute to the subject’s dietary regime by proposing nutritional interventions that would reverse gut microbial dysregulation.

## Methods

### Study population

A case–control study was conducted with subjects 7 to 17 years-old from an obesity clinic of a tertiary care hospital (*Hospital Regional Materno Infantil de Alta Especialidad*) in Nuevo León, Mexico between 2016 and 2017. A sample of 21 subjects with T2DM, 25 with MetS, and 20 controls (*n *= 66) were studied. Inclusion criteria for T2DM were according to the American Diabetes Association [54] and MetS was diagnosed to subjects with at least three out of the five criteria established by Cook *et al *[55]. Even though Cook’s et al. criteria were originally established for adolescents, they are widely used among school-aged children as a current consensus for MetS in children is lacking. Control subjects were individuals that presented no signs of T2DM and MetS and considered healthy by their pediatrician.

Exclusion criteria considered subjects younger than 7 years and older than 17 years, with irritable bowel syndrome and/or ulcerative colitis, history of diarrhea within the last 3 months, disease that affects the intestinal motility (hyperthyroidism, hypothyroidism), atopic dermatitis and that have used probiotics, prebiotics, antibiotics, or medication that affects intestinal motility within the last 3 months. Elimination criteria also considered subjects with incomplete information. Subjects that fulfilled inclusion criteria as cases and controls were invited personally during a visit to the obesity clinic or pediatrician, respectively. Once the subjects agreed to participate, informed consent was obtained from their parents or legal tutors as well as from the subjects. This study was conducted according to the ethical principles of the Declaration of Helsinki and approved by the Ethics and Research Committees of the School of Medicine *Tecnologico de Monterrey* and the government state education authorities of Nuevo León, Mexico. The study was approved by the ethics committees and institutional review boards of Hospital Regional Materno Infantil and the School of Medicine *Tecnologico de Monterrey* (CONBIOETICA19CEI00820130520) and was authorized by the Federal Commission for the Protection against Sanitary Risks (COFEPRIS) (13CE/19039139 and 13CE/19039138).

### Anthropometric and biochemical parameters

Anthropometric and biochemical variables were evaluated using Spearman correlation to identify potential associations with the gut bacterial communities at genus level and linear discriminant analysis (LDA) was conducted at family and genus levels as a first attempt to use gut bacteria as potential biomarkers for their further use in the prediction of the studied health conditions within pediatric subjects.

Anthropometric measurements were obtained following the National Health and Nutrition Examination Survey (NHANES) criteria, wearing lightweight clothing and without shoes. Weight and height were determined using a TANITA BF689® scale and SECA® mechanical wall mount stadiometer, respectively. Body mass index (BMI) was calculated using the Quetelet formula and was classified by percentiles for age and sex according to the normative charts from *LMS Parameters for Boys and Girls: BMI for Age,* NHANES*,* CDC-2000 [56].

Waist circumference was measured using a SECA® fiberglass measuring tape with the subject standing, at the end of a normal expiration, midway between the lowest rib and iliac crest. Systolic and diastolic blood pressure (BP) were determined by standardized personnel with a mercury sphygmomanometer and an age-appropriate bracelet, covering more than 50% of the arm circumference. Three measurements were performed within 15 min intervals. BP percentiles for sex, age and height were obtained according to the *National High Blood Pressure Education Program Working Group on High Blood Pressure in Children and Adolescents *[57]*.*

Peripheral blood was also obtained from all the studied subjects through venipuncture after 8 h overnight fasting. Samples were stored at 2—8ºC and transported to the laboratory for processing. Samples were centrifuged within the first 3 h to retrieve serum and plasma which were stored at -80 °C until determination of biochemical measurements consisting of total cholesterol (TC), triglycerides (TG), high-density lipoprotein (HDL-c), low-density lipoprotein (LDL-c), and glucose. Additionally, information regarding medications taken by the studied individuals was also recorded.

### Stool sample collection

Fecal samples were collected and stabilized using OMNIgene®-GUT OMR 200 kit (DNA Genotek Inc., Ottawa, Ontario, CA). A small amount of sample of each studied subject was collected using the spatula contained in the kit and transferred to the kit’s tube. The sample was mixed with the stabilizing solution in a back-and-forth motion for at least 30 s. Sealed tubes were stored at room temperature and transported to the laboratory for processing.

### DNA extraction

FastDNA® Spin Kit for Feces (MP Biomedicals, California, USA) was used for DNA extraction following manufacturer’s instructions. Cell lysis was performed using the FastPrep-24™ Homogenizer (MP Biomedicals, California, USA). Sample DNA concentration and purity was evaluated using a Nanodrop™ Spectrophotometer (Thermo Scientific, Delaware, USA). Extracted DNA was stored at -20 ºC until further analysis.

### Library preparation and sequencing

The 16S Metagenomic Sequencing Library Preparation protocol from Illumina was used to prepare the sequencing library based on the V3-V4 variable regions of the 16S rDNA gene. Quality and concentration of the samples were evaluated using the Qubit 2.0 Fluorometer (Invitrogen, Massachusetts, USA) and 2100 Bioanalyzer (Agilent Genomics, California, USA). Libraries were sequenced (2 × 300 bp paired-end) with the MiSeq Reagent Kit V3 (600 cycles) using the Illumina MiSeq system (Illumina, California, USA) at the sequencing facilities of Tecnologico de Monterrey (Monterrey, Mexico). All sequences have been archived in the NCBI Sequence Read Archive under BioProject no. PRJNA819279 (https://www.ncbi.nlm.nih.gov/bioproject/?term=PRJNA819279).

### Bioinformatic and statistical analyses

Sequence raw reads were analyzed using QIIME v1.9.1 [58]. following a standard 16S rDNA analysis pipeline, with a similarity threshold of 97% and using GreenGenes 13_8 as database reference for an open-reference approach to retrieve information from taxonomic classification reported as operational taxonomic units (OTUs). For this, low-quality sequences were trimmed and excluded. Later, data were processed to remove rare microorganisms (≤ 1% read relative abundance of each sample) and they were normalized using a variance transformation approach with *DESeq2* package [59] from RStudio software version 4.1.2 [60].

Homoscedasticity and normality tests were conducted for anthropometric, demographic, clinical, and biochemical characteristics (subjects’ metadata). Subsequently, microbial diversity was explored through α- and β-diversity analyses. Richness, Chao1, Shannon, and Simpson indices were obtained for α-diversity and a principal coordinate analysis (PCoA) was conducted to determine differences among bacterial communities at phylum, family, and genus levels (β-diversity). The subjects’ metadata and its possible correlation with gut microbiota composition was analyzed using Spearman correlation and a subsequent p-value correction using the Benjamini–Hochberg procedure. Rarefaction curves were calculated using the raremax function to determine the minimum number of reads per sample, followed by the rrarefy function.

The *vegan *[61]*, agricolae *[62], *FSA *[63], *rcompanion *[64], *car *[65], and *ggcorrplot *[66] packages in RStudio were used for statistical analyses. Barplots and boxplots were constructed using *ggplot2 *[67] and *patchwork *[68], where read relative abundance ≤ 1% was marked as “Others”.

Finally, a linear discriminant analysis (LDA) [69] was conducted to classify each of the studied subjects within categorical dependent groups (based on their health condition) using linear combinations of microbiome-based predictors, maximizing the between-group variance, and comparing it to the within-group variance. The bacterial communities at family and genus levels used for this analysis were managed with an assumption of normality in their abundance, conducting one-way analysis of variance (ANOVA) to select those who displayed a significant difference among health conditions using p-value ≤ 0.05. These p-values were corrected using the Benjamini–Hochberg procedure. An absolute value of their standardized coefficients ≥ 1 retrieved from LDA was used to determine their relevant contribution to the studied groups (MetS, T2DM, and controls). P-values are obtained for one way ANOVA, which is a univariate analysis. LDA is a multivariate analysis used to determine which microorganisms have a higher contribution in the studied groups compared to all microorganisms in the consortia. LDA coefficients will define which microorganisms are better to distinguish or are associated with a certain condition. These statistical analyses were also performed with RStudio using the *MASS *[70] and *car *[65] packages.

## Supplementary Information

**Additional file 1: Table S1.*** Cardiometabolic risk factors in subjects with T2DM, MetS and controls.*** Table S2.*** Medical treatments taken by studied subjects with T2DM, MetS and controls.*** Table S3.*** Sequencing statistics of samples used in the study.*** Table S4. ***Operational taxonomic units (OTUs) from the studied groups.*** Table S5. ***Operational taxonomic units (OTUs) at phylum level.*** Table S6. ***Operational taxonomic units (OTUs) at family level.*** Table S7. ***Operational taxonomic units (OTUs) at genus level.*** Table S8. ***Read relative abundance of bacterial communities at genus level > 1% within each studied group.*** Table S9. ***Linear discriminant analysis of relevant microorganisms at family level.*** Table S10. ***Linear discriminant analysis of relevant microorganisms at genus level.*

**Additional file 2: Figure S1.*** Rarefaction curves at genus level from control, MetS, and T2DM study groups*. *(****A****)* *Per studied group;* *(****B****)* *Per studied sample.*** Figure S2.** *Alpha diversity at species level in the control, MetS, and T2DM study groups. (****A****) Richness; (****B****) Chao1 Index; (****C****) Shannon Index; (****D****) Simpson Index. Tukey HSD (Richness and Chao1 Index) and Kruskall-Wallis (Shannon and Simpson Index) tests were conducted to study differences among study groups.*** Figure S3. ***Relative read abundance (****A****) and clustering of communities by PCoA using Bray-Curtis dissimilarity matrix as distance (****B****) of gut microbiota phyla. Data represent relative abundances greater or equal to 1%. Each dot corresponds to a community in a specific group.*** Figure S4.** *Read relative abundance* *(****A****)* *and clustering by PCoA* *(****B****)* *of gut bacterial families.* *Data represent read relative abundances greater or equal to 1%.* *Each dot corresponds to an individual’s bacterial community in a specific group.*** Figure S5. ***Linear discriminant analysis (LDA) from gut bacterial communities at genus level from the studied population.*

## Data Availability

16S rDNA gene sequences from each participant have been archived in the NCBI Sequence Read Archive under BioProject accession no. PRJNA819279 (https://www.ncbi.nlm.nih.gov/bioproject/?term=PRJNA819279).

## Abbreviations

BMI: Body mass index
BP: Blood pressure
HDL-c: High-density lipoprotein
IR: Insulin resistance
LDA: Linear discriminant analysis
LDL-c: Low-density lipoprotein
MetS: Metabolic syndrome
NHANES: National Health and Nutrition Examination Survey
OUT: Operational taxonomic unit
TC: Total cholesterol
TG: Triglycerides
T2DM: Type-2 diabetes mellitus
