# Supplementary material for: Characterization of gut microbiota associated with metabolic syndrome and type-2 diabetes mellitus in Mexican pediatric subjects
Source: BMC Pediatr. 2023 May 3;23:210. doi: 10.1186/s12887-023-03983-6 (PMC10155456; doi:10.1186/s12887-023-03983-6)
Supplement: Supplementary file 2 — Additional file 2: Figure S1. Rarefaction curves at genus level from control, MetS, and T2DM study groups. (A) Per studied group; (B) Per studied sample. Figure S2. Alpha diversity at species level in the control, MetS, and T2DM study groups. (A) Richness; (B) Chao1 Index; (C) Shannon Index; (D) Simpson Index. Tukey HSD (Richness and Chao1 Index) and Kruskall-Wallis (Shannon and Simpson Index) tests were conducted to study differences among study groups. Figure S3. Relative read abundance (A) and clustering of communities by PCoA using Bray-Curtis dissimilarity matrix as distance (B) of gut microbiota phyla. Data represent relative abundances greater or equal to 1%. Each dot corresponds to a community in a specific group. Figure S4. Read relative abundance (A) and clustering by PCoA (B) of gut bacterial families. Data represent read relative abundances greater or equal to 1%. Each dot corresponds to an individual’s bacterial community in a specific group. Figure S5. Linear discriminant analysis (LDA) from gut bacterial communities at genus level from the studied population. [file 12887_2023_3983_MOESM2_ESM.pdf]

Supplementary Material

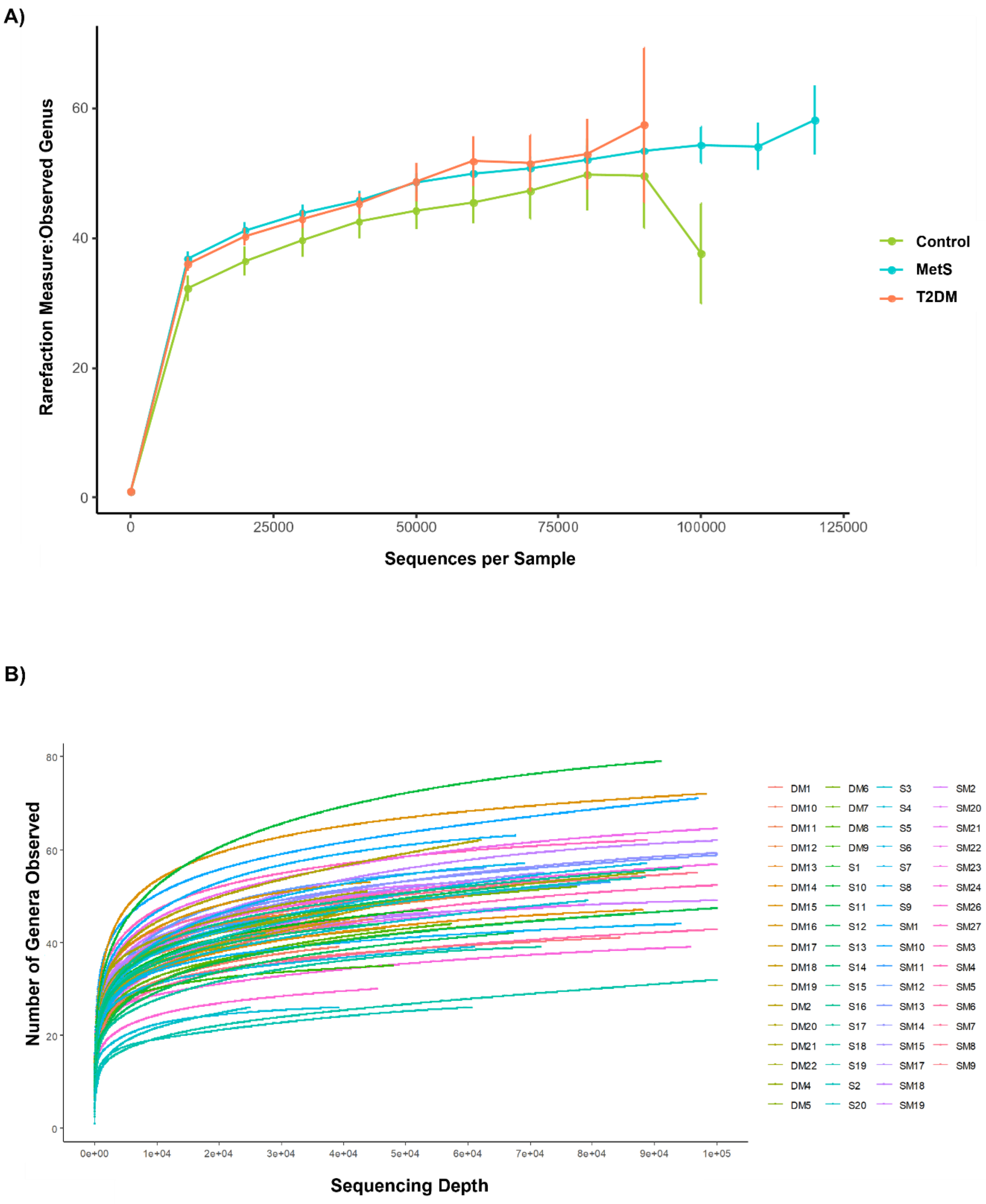

Figure S1. Rarefaction curves at genus level from control, MetS, and T2DM study groups. (**A)** Per studied group; (**B)** Per studied sample

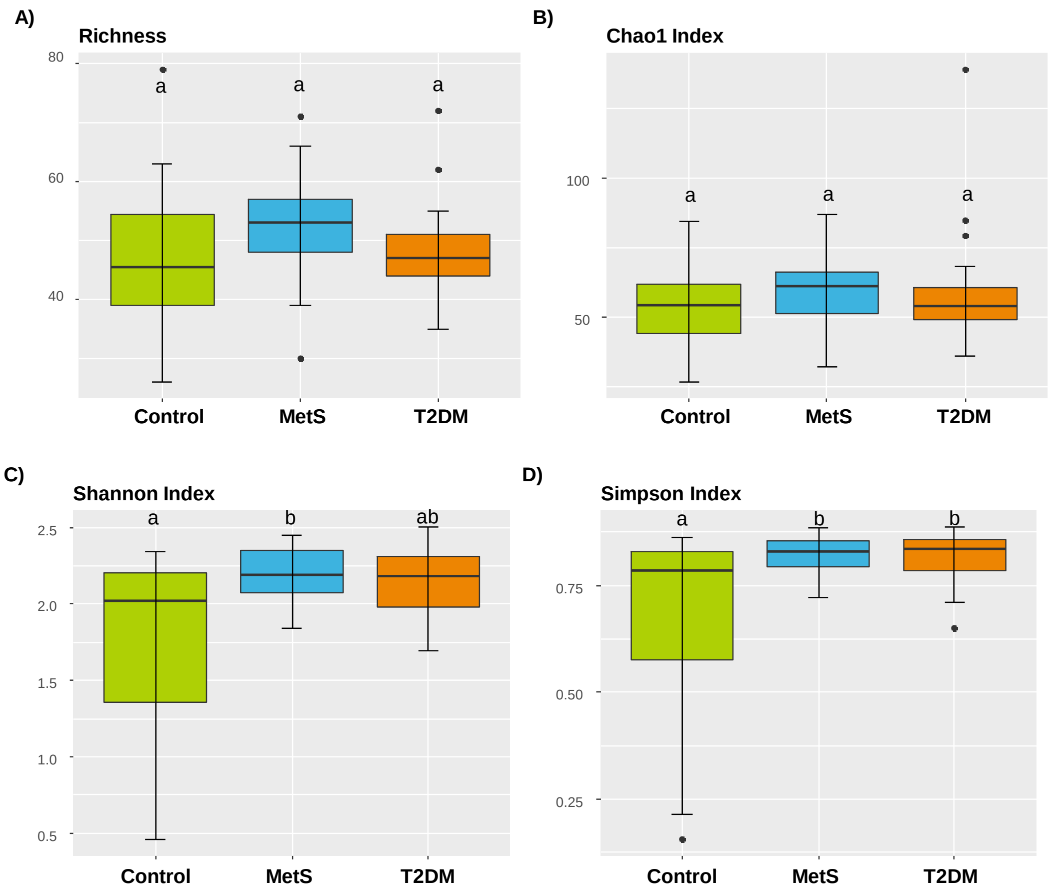

Figure S2. Alpha diversity at species level in the control, MetS, and T2DM study groups. (**A)** Richness; (**B)** Chao1 Index; (**C)** Shannon Index; (**D)** Simpson Index. Tukey HSD (Richness and Chao1 Index) and Kruskall-Wallis (Shannon and Simpson Index) tests were conducted to study significant differences between study groups: Shannon Index was significantly different between MetS ("b") and Control group ("a"). Simpson Index in MetS and T2DM groups ("b") resulted to be significantly different compared to the Control group ("a").

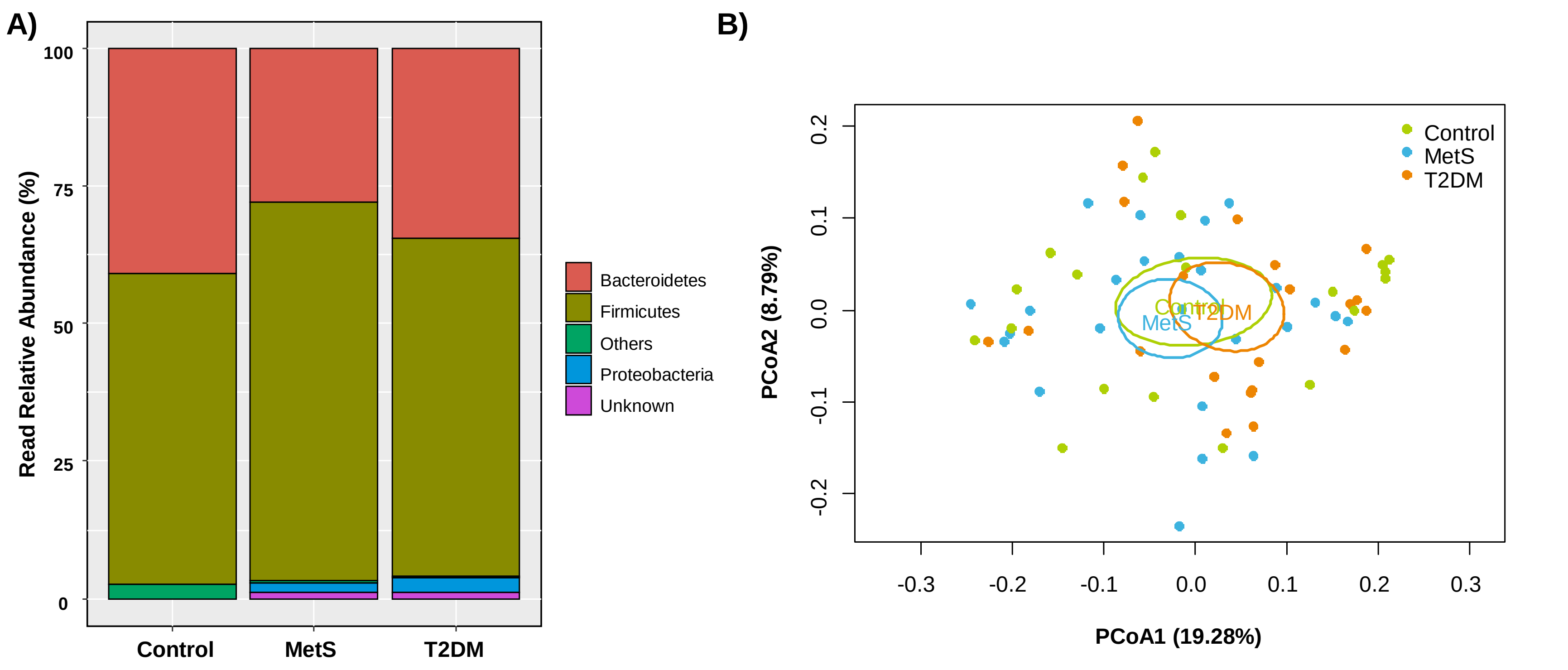

Figure S3. Relative read abundance **(A)** and clustering of communities by PCoA using Bray-Curtis dissimilarity matrix as distance **(B)** of gut microbiota phyla. Data represent relative abundances greater or equal to 1%. Each dot corresponds to a community in a specific group.

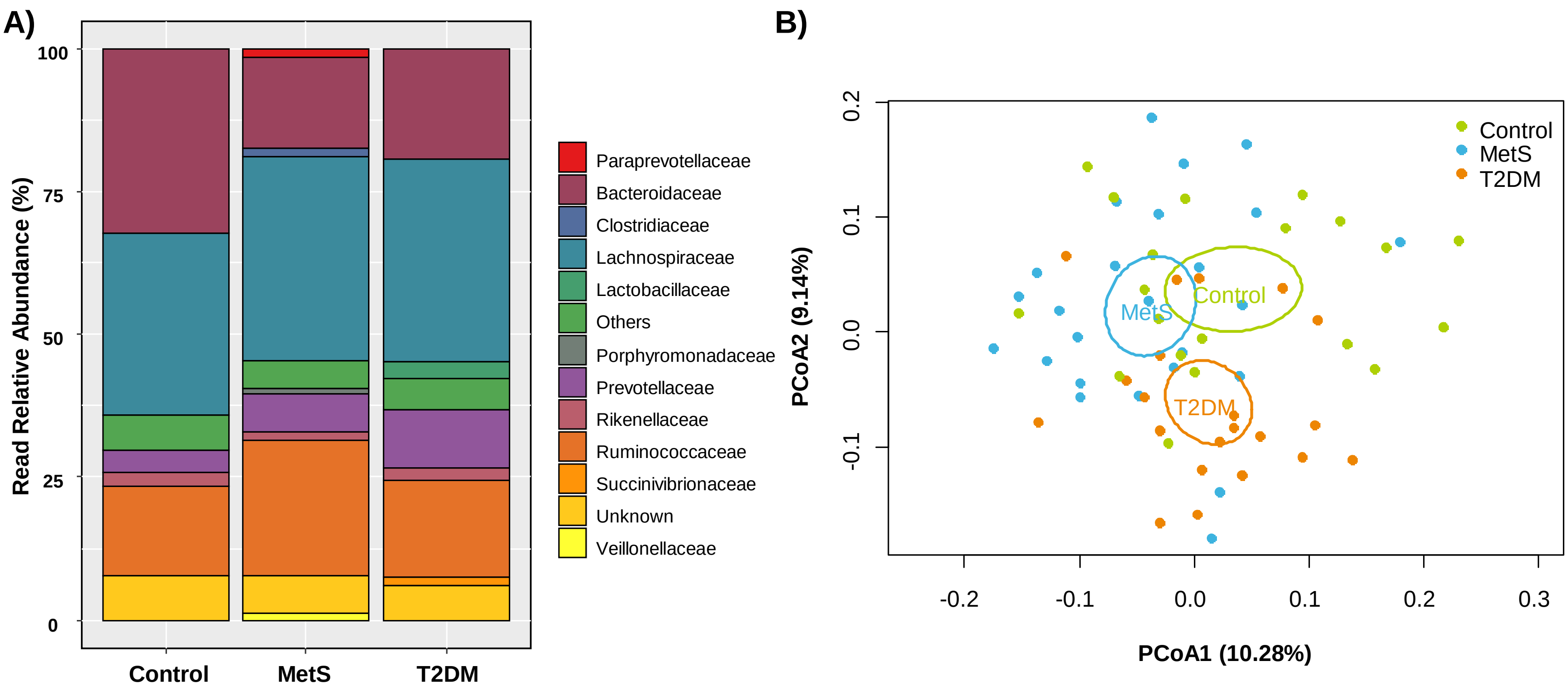

Figure S4. Read relative abundance (A) and clustering by PCoA (B) of gut bacterial families. Data represent read relative abundances greater or equal to 1%. Each dot corresponds to an individual's bacterial community in a specific group.

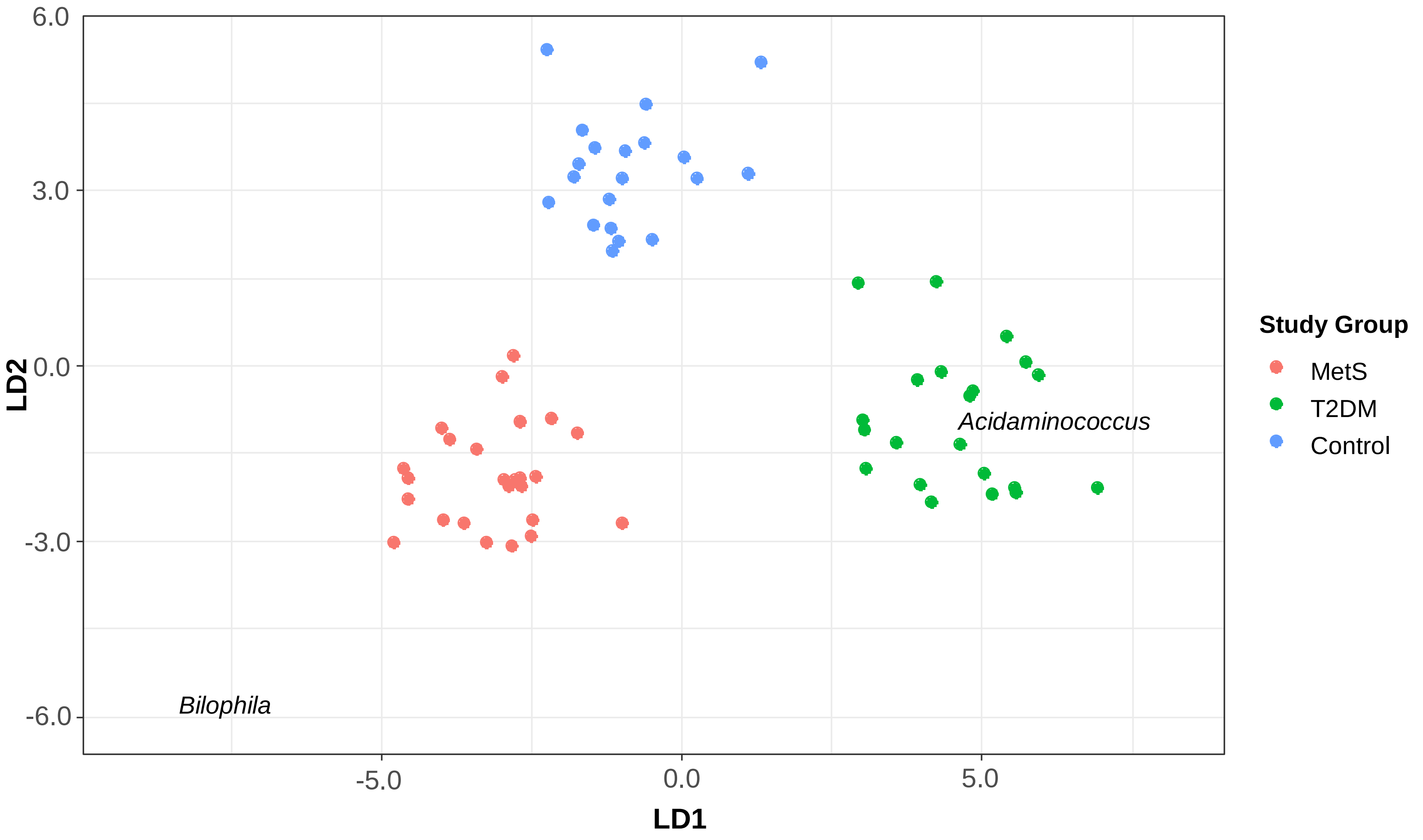

Figure S5. Linear discriminant analysis (LDA) from gut bacterial communities at genus level from the studied population.
